# Preoperative evaluation of alcohol consumption in older patients

**DOI:** 10.1186/s13722-025-00569-8

**Published:** 2025-05-21

**Authors:** Vera Guttenthaler, Maria Wittmann, Jan Menzenbach

**Affiliations:** Clinic for Anesthesia and Intensive Care Medicine, University Hospital Bonn, Venusberg-Campus 1, 53127 Bonn, Germany

**Keywords:** Alcohol consumption, AUDIT-C, Anesthesiological evaluation, Preoperative alcohol assessment

## Abstract

**Purpose:**

This sub-analysis of the PROPDESC-study (Pre-Operative Prediction of postoperative delirium by appropriate Screening-study) evaluated the alcohol consumption of older patients with two different assessment tools (single sentence question and Alcohol Use Disorder Identification Test-Consumption (AUDIT-C)) and compared the results in regards to detection, reliability, and quantification of patient´s alcohol consumption.

**Methods:**

During their anesthesiological pre-clinic visit 1084 patients older than 59 years were asked whether they consume alcohol daily and 668 of them additionally answered the AUDIT-C questionnaire.

**Results:**

According to the SSQ 11.72% of the patients consumed alcohol daily. In the AUDIT-C sub-group 25.90% reported moderate to high alcohol consumption while infrequent or very low alcohol intake was reported by 41.92%. In the subgroup 31.89% of the patients stated alcohol abstinence. About one quarter (25.13%) of patients who denied daily alcohol intake but scored positive on the AUDIT-C displayed levels of alcohol consumption ranging from moderate (11.20%) to high (13.87%) according to the AUDIT-C.

**Conclusion:**

Reliable information about alcohol consumption is related to the method of questioning. The AUDIT-C evaluates the patient´s alcohol intake precisely and identifies more older patients with possibly health- and surgery-relevant alcohol consumption levels. The validated AUDIT-C provides an objective assessment to the physician during the pre-clinic anesthesiologic consultation. Additionally, handing out a questionnaire to the patient encourages initiative and self-assessment and could also relieve both, the physician and the patient from sensing a moral evaluation of alcohol consumption.

**Supplementary Information:**

The online version contains supplementary material available at 10.1186/s13722-025-00569-8.

## Introduction

According to the 2024 World Health Organization (WHO) Report on alcohol consumption the level of “per capita alcohol consumption” (APC) in the European Union (EU) is still the highest in the world [1]. A nationwide representative survey of 18- to 64-year-old German citizens that used the AUDIT (Alcohol Use Disorder Identification Test) as assessment tool revealed that 70.5% had consumed alcohol in the past 30 days [2] with episodes of binge drinking being more often reported by men (41.9%) than by women (23.3%) [2]. Problematic alcohol consumption (defined as exceeding an average daily consumption of pure alcohol of 12 g for women or 24 g for men) was present in 17.6% of respondents [2].

Therefore, patients who are scheduled for elective surgery in a German hospital have a reasonable high possibility to present with a history of alcohol consumption.

In individuals with excessive alcohol consumption, the manifestation of alcohol withdrawal symptoms during their hospital stay could pose significant life-threatening dangers [3, 4]. These risks stem on one hand from interactions between medications as well as direct pharmacological interactions between alcohol and narcotics and on the other hand from physiological alterations [3, 4]. Therefore, it is crucial to detect patients at risk for alcohol withdrawal syndrome (AWS) as early as possible in order to treat them with the appropriate prophylactic medication [5].

The underlying causes of a heightened perioperative risk for high level alcohol drinkers likely involve multiple factors, such as dysfunction of various organ systems induced by alcohol before surgery, an amplified response to surgical stress, and/or dysfunctions triggered by abstinence [6]. Research on pathophysiological mechanisms indicates that excessive alcohol intake diminishes immune function, heightens the endocrine stress response to surgery, and retards the wound healing process [7]. In total joint arthroplasty the amount of alcohol intake of male patients could influence the number of postoperative complications [8] and male patients diagnosed as chronic alcohol drinkers that had to undergo major tumor surgery had an increased risk for mortality and morbidity after surgery [9].

A meta-analysis by Eliasen et al. revealed that preoperative alcohol consumption correlates with heightened risks of a variety of postoperative complications, encompassing general morbidity, wound healing problems, pulmonary complications, prolonged hospital stay, and intensive care unit admission [10]. The same review found no association between low to moderate alcohol consumption and general morbidity and infections, wound intricacies, and cardiopulmonary or neurological complications [10]. Eliasen et al. additionally conducted a sub-analysis to evaluate the impact of high alcohol consumption and their findings indicated that excessive alcohol intake was also linked to a heightened risk of postoperative mortality [10]. The nature of the surgical procedure did not appear to affect the relationship between alcohol consumption and postoperative complications [10]. Rubinsky et al. evaluated that surgical patients with very high AUDIT-C scores (9–12 points) stayed longer on ICU (intensive care unit) and in hospital, and had an increased risk of return to the operating room within 30 days after their surgery compared to the low-risk drinking patients (AUDIT-C scores 1–4) [11].

To be able to estimate a possible risk of alcohol-induced perioperative complications provides the opportunity to initiate measures in a timely manner. Therefore, reliable estimation of a patient´s alcohol consumption is of high importance for the attending physicians.

However, patients seem to expect discussions about their alcohol consumption more during appointments or routine check-ups than during consultations for non-alcohol-related issues [12]. Another barrier to effective screening before a surgical intervention could be insufficient management of staff workload or a reluctance of the professional to ask patients about alcohol drinking without clear signs of risky drinking behavior [12]. Avoidance of questions about alcohol consumption levels could be related to the fact, that some professionals had encountered negative reactions from the respondent in terms of embarrassment and unease, which emphasizes that a good rapport between patient and professional is helpful in discussing sensitive topics such as drinking behavior [12].

The U.S. Preventive Service Task Force (ASPSTF) recommends using the NIAAA (National Institute on Alcohol Abuse and Alcoholism) Single Alcohol Screening Question (SASQ) or the AUDIT-C as quick and effective screening tools [13]. The SASQ is part of a two-step screening process. Initially, participants are asked about their occasional consumption of alcoholic beverages, and only those who respond affirmatively are asked the subsequent screening question: “How many times in the past year have you had X or more drinks in a day?” (where “X is 5 for men and 4 for women, and a response of > 1 is considered positive”) [13] In 2009, Smith et al. conducted a trial with 286 subjects, the majority of them identifying themselves as black or African-American [14]. He compared the two steps screening process mentioned above with the AUDIT-C and evaluated that this face-to-face questioning demonstrated comparable sensitivity and specificity in identifying unhealthy alcohol use among his sample of primary care patients compared to the AUDIT-C [14]. The AUDIT-C itself has been validated in various settings and cohorts. Bradly et al. found in a study with adult outpatients at an academic family practice clinic that the AUDIT-C performed as well as the full AUDIT and significantly better than self-reported risky drinking [15]. Bush et al. interviewed 393 male general medical patients and found the questions of the AUDIT-C to be a practical, valid primary care screening test for heavy drinking and/or dependence [16].

The objective of this sub-analysis was to assess the reliability of a SSQ (Single sentence question) administered in a pre-clinical context compared to the outcomes obtained from the Alcohol Use Disorders Identification Test Consumption questions (AUDIT-C) concerning the identification and measurement of preoperative alcohol intake.

## Methods

Results of this analysis are derived out of the PROPDESC-study (Pre-operative prediction of postoperative delirium by appropriate screening-study) that was designed and conducted as an prospective, observational, mono-centric study to create an easy pre-operative score to detect patients at risk for postoperative delirium [17, 18]. Inclusion criteria for the PROPDESC study were age of 60 years or older, a planned surgical intervention of more than 60 min, and written informed consent. Patients undergoing emergency procedures, patients with difficulties in the German language or pre-existing mental retardation or severe dementia that might complicate cognitive testing and delirium assessment were excluded. Laboratory values that have been recognized as possible predictive values for the occurrence of POD (postoperative delirium) were assessed in the cause of pre-clinic routine laboratory blood examination [17]. Since heavy alcohol intake was recognized as a risk factor for postoperative delirium [19], all participants were questioned about their daily alcohol consumption habits by trained study personnel during their visit to the anesthesiological pre-admission clinic. Further details of the PROPDESC study can be found in the publication of the study protocol [17].

After the inclusion of 429 patients, an amendment was made to the study, that added the conduction of the AUDIT-C. The AUDIT-C questionnaire comprises the first three questions of the AUDIT [20]. The first question asks about the frequency of consuming alcoholic beverages. The second question inquires the typical number of standard drinks consumed per day. The third question focuses on the frequency of consuming six or more drinks on a single occasion [20]. As mentioned above the AUDIT-C was validated in various settings as a screening tool for heavy alcohol consumption.

Answers to the AUDIT-C are rated 0–4 points, adding up to a maximum of 12 points. In our analysis four groups of consumption levels were distinguished: no consumption/abstinence (0 points), low or infrequent (1–3 points), moderate (4 points), and high (AUDIT-C score > 4) alcohol consumption in the year before the questioning. These groups were formed under the consideration that the cut-off value for heavy alcohol consumption has to be adjusted to the age to achieve reasonable sensitivity and specificity of the results. Aalto et al. found that a cut-off of > = 4 led to a sensitivity of 0.94 and a specificity of 0.80 for the detection of heavy drinking in a stratified random sample of 804 Finns aged 65–74 years [21]. The AUDIT has been translated into many languages. All translations are available online [22]. In this setting the German version of the AUDIT-C version recommended by the German Medical Association (Bundesärztekammer, Suchtforschungsverbund Baden-Württemberg, UKL Freiburg) was used [23]. This version defined one glass of alcohol as the equivalent of 0.33 L beer, 0.25 L wine of sparkling wine or 0.02 L of liquor. After approval of the protocol amendment study patients were additionally asked the AUDIT-C regardless of the answer given to the SSQ question. All study related procedures were conducted by trained study personnel.

Statistics: Answers to the questions about alcohol consumption were evaluated in the complete patient group and for female and male patients separately. The level of alcohol consumption in relation to age was examined by dividing the AUDIT-C groups in subgroups spanning 10-year increments. Alcohol consumption was analyzed in the total patient group and in the group of patients that additionally answered the AUDIT-C questionnaire. Statistical analysis was performed using the statistical programming environment R. For the description of the cohorts, continuous and ordinal variables are presented with mean and ± standard deviation (sd). Nominal variables are reported as numbers and percentages. Laboratory values are presented with median and inter-quartile range (IQR), due to the inherent skewness. The differences between the cohorts were analyzed using the nonparametric Wilcoxon rank sum test for continuous variables (no normal distribution was present) and Fisher’s exact test for categorical variables, using a two-sided significance level of 0.05.

## Results

**Fig. 1:**
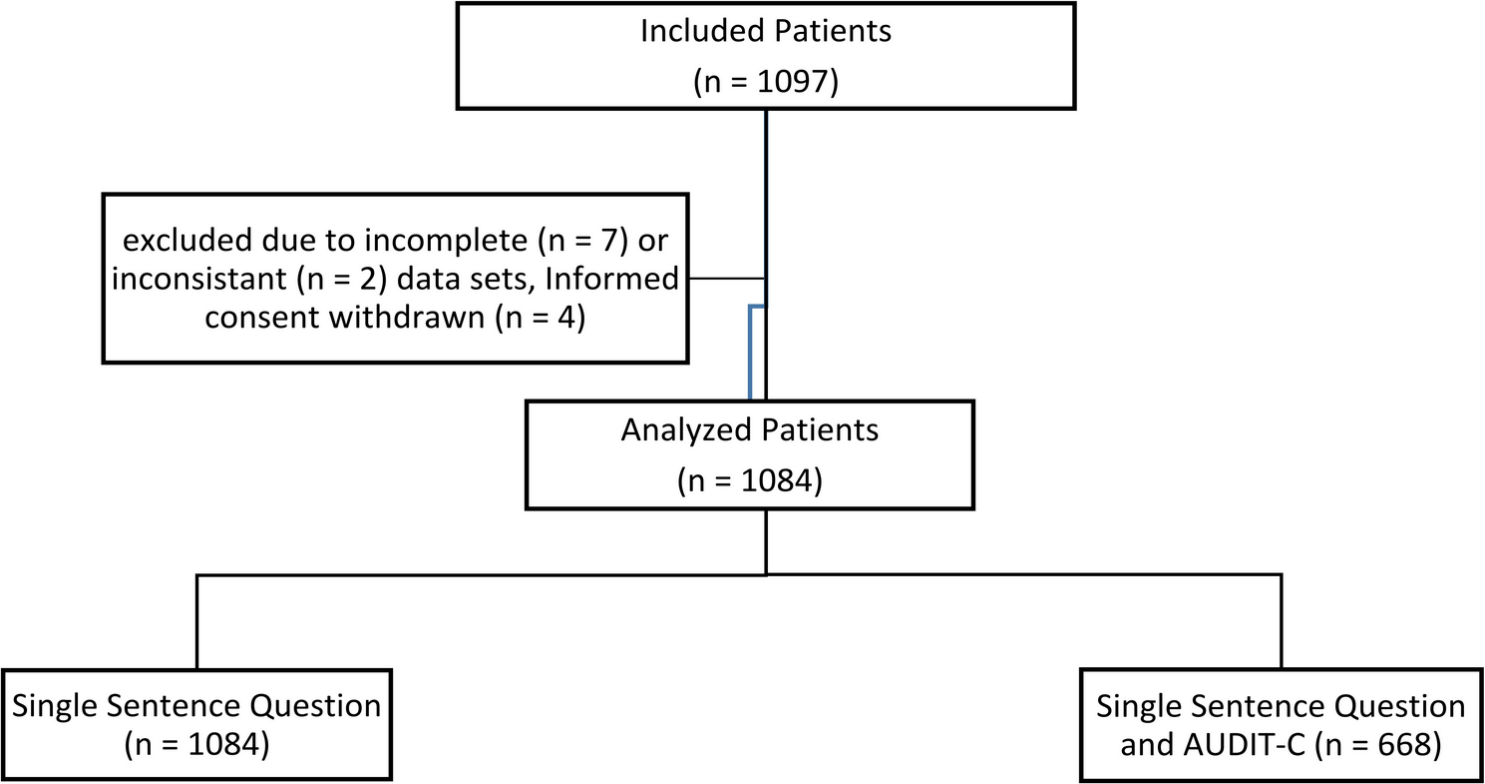
Modified after Menzenbach et al. [18]

Of the 1097 included patients seven patients were excluded due to missing answers to the SSQ. Additionally, four patients were excluded, because they withdrew their informed consent to the PROPDESC study and two patients were excluded due to inconsistent data (Fig. 1).

Therefore, 1084 patients that answered the SSQ were analyzed; 425 (39.28%) of them were women. Patients had a mean age of 72.42 years (Table 1). The patient sample included 668 patients that additionally answered the AUDIT-C. Of all included patients 957 (88.28%) answered the SSQ in the negative and 127 (11.72%) confirmed daily alcohol (Table 1). The characteristics of the patients that answered the SSQ negative differed not significantly from the daily drinkers in regards to their age, ASA (American Society of Anesthesiologists) class and NYHA (New York Heart Association) classification, their planned surgical category, and their revised cardiac risk index (rCRI). Daily drinking patients differed in some aspects significantly from patients that negated daily alcohol intake. In the group confirming daily alcohol consumption were significantly more men (*p* < 0.001), patients had a significantly higher education (*p* < 0.001), and scored significantly higher on the MoCA (Montreal Cognitive assessment) test (*p* = 0.017) (Table 1). They also had significantly higher gamma-GT (gamma glutamyl transpeptidase) laboratory values (*p* < 0.001) and hemoglobin values (*p* = 0.036), but it has to be noted that many laboratory values are missing as they were not assessed routinely at the pre-admission visit (Table 1).

**Table 1:** Comparison of all patients in regards to their answer to the SSQ

	All patients	SSQ negativ	SSQ positiv	*p*-value	missings
N (%)	1084 (100.00)	957(88.28)	127 (11.72)		
Age(mean ± sd)	72.42 (± 7.4)	72.53 (± 7.35)	71.65 (± 7.76)	0.164	0
Sex				< 0.001*	2
Female, n (%)	425	403 (42.11)	22 (17.32)		
Male, n (%)	657	552 (57.68)	105 (82.69)		
ASA, n (%)				0.634	9
1	26	23 (2.4)	3 (2.36)		
2	364	318 (33.23)	46 (36.22)		
3	600	530 (55.38)	70 (55.12)		
4	84	78 (8.15)	6 (4.72)		
5	1	1 (0.1)	0		
Surgical department, n (%)				0.703	
cardiac surgery	305	275 (28.74)	30 (23.62)		
Thoracic surgery	23	21 (2.19)	2 (1.57)		
Abdominal surgery	144	124 (12.96)	20 (15.75)		
Vascular surgery	31	27 (2.82)	4 (3.15)		
Orthopedic surgery	365	324 (33.86)	41 (32.28)		
others	216	186 (19.44)	30 (23.62)		
rCRI, n (%)				0.070	12
rCRI 1	440	387 (40.44)	53 (44.88)		
rCRI 2	268	230 (24.03)	38 (29.92)		
rCRI 3	236	209 (21.84)	27 (21.26)		
rCRI 4	128	121 (12.64)	7 (5.51)		
NYHA				0.231	14
NYHA I	445	388 (40.54)	57 (44.88)		
NYHA II	367	320 (33.44)	47 (37.01)		
NYHA III	236	217 (22.68)	19 (14.96)		
NYHA IV	22	20 (2.09)	2 (1.57)		
MoCA Sum(mean ± sd)	23.00 (± 3.94)	22.90 (± 3.94)	23.77(± 3.86)	0.017*	0
MoCA Education				< 0.001*	3
> 12 years	609	513 (53.61)	96 (75.59)		
≤ 12 years	473	443 (46.29)	30 (23.62)		
Laboratory values (median) [IQR]					
GLDH 37 C (U/l)	3.3 [2.25]	4.77 [5.1]	4.21 [3.2]	0.856	961
ALT(GPT) 37 C (U/l)	23 [16]	28 [20.64]	40.08 [56.77]	0.276	661
AST(GOT) 37 C (U/l)	25 [11]	31.19 [32.98]	28.02 [12.49]	0.675	669
gamma-GT 37 C (U/l)	34 [50]	75.16 [180.12]	133.93 [215.03]	< 0.001*	767
alk.Phosphatase 37 C (U/l)	79.5 [44.25]	113.87 [117.15]	141.23 [150.69]	0.477	992
Hemoglobine (g/dl)	13.4 [2.3]	13.17 [1.88]	13.53 [1.87]	0.036*	2
Creatinine (mg/dl)	0.9 [0.35]	1.06 [0.73]	1.01 [0.44]	0.781	2
CRP (mg/l)	3.26 [7.73]	12.15 [28.28]	9.36 [17.3]	0.828	9
Total proteine (g/l)	69.2 [6.9]	68.54 [6.02]	69.42 [5.58]	0.238	23

N = number, SSQ = single sentence question, *=significant with p-value < 0.05, sd = standard deviation, ASA = American Society of Anesthesiologist Score, rCRI = revised cardiac risk index, NYHA = New York Heart Association Score, MoCA (Montreal Cognitive Assessment), IQR = interquartile range, GLDH = Glutamate dehydrogenase, ALT = Alanine aminotransferase, AST = Aspartate aminotransferase, GGT = gamma glutamyl transpeptidase, alk. Phosphatase = alkaline phosphatase, CRP = C-reactive protein

To the SSQ question 668 patients additionally answered the AUDIT-C. Of those patients 173 (total 25.90%, with 21.71% men and 4.19% women) scored more than 3 points on the AUDIT-C indicating moderate to high alcohol consumption. Table 2 shows the characteristics of the AUDIT-C group and the subgroups comprised of patients who scored 0–3 points and patients with more than 3 points on the AUDIT-C. Patients in the higher consumption group were significantly younger (*p* = 0.008), had a higher education (*p* = 0.003), and scored better on the MoCA test (Montreal Cognitive Assessment) than patients with an AUDIT-C result below 4 points. The differences in the laboratory values matches those of the SSQ group. Patients with AUDIT-C results above the Cut-off had significantly higher gamma-GT values (*p* = 0.014) and significantly higher hemoglobin values (*p* = 0.003) (Table 2).

**Table 2:** Comparison of all patients that answered the AUDIT-C

	All AUDIT-C patients	AUDIT-C 0–3	AUDIT-C > 3	*p*-value	missings
Number	668	495	173		
Age(mean ± sd)	72.23 (± 7.39)	72.69 (7.37)	70.92 (7.34)	0.008*	0
Sex				< 0.001*	2
Female, n (%)	250 (37.54)	222 (44.85)	28 (16.18)		
Male, n (%)	416 (62.46)	271 (54.75)	145 (83.82)		
ASA, n (%)				0.372	9
1	17 (2.58)	12 (2.42)	5 (2.89)		
2	195 (29.59)	139 (28.08)	56 (32.37)		
3	395 (59.94)	295 (59.60)	100 (57.80)		
4	51 (7.74)	43 (8.69)	8 (4.62)		
5	1 (0.15)	1 (0.20)	0 (0.00)		
Surgical department, n (%)				0.286	0
cardiac surgery	228 (34.13)	177 (35.76)	51 (29.48)		
Thoracic surgery	17 (2.54)	13 (2.63)	4 (2.31)		
Abdominal surgery	78 (11.68)	58 (11.72)	20 (11.56)		
Vascular surgery	18 (2.69)	13 (2.63)	5 (2.89)		
Orthopedic surgery	201 (30.09)	151 (30.51)	50 (28.90)		
others	126 (18.86)	83 (16.77)	43 (24.86)		
rCRI, n (%)				0.057	11
rCRI 1	256 (38.96)	190 (38.38)	66 (38.15)		
rCRI 2	161 (24.51)	113 (22.83)	48 (27.75)		
rCRI 3	157 (23.90)	114 (23.03)	43 (24.86)		
rCRI 4	83 (12.63)	71 (14.34)	12 (6.94)		
NYHA, n (%)				0.668	13
NYHA I	259 (39.54)	187 (37.78)	72 (41.62)		
NYHA II	234 (35.73)	173 (34.95)	61 (35.26)		
NYHA III	150 (22.90)	116 (23.43)	34 (19.65)		
NYHA IV	12 (1.83)	10 (2.02)	2 (1.16)		
MoCA Sum(mean ± sd)	23.43 (± 3.86)	23.2 (± 3.9)	24.09 (± 3.69)	0.011*	
MoCA Education				0.003*	
> 12 years	389 (58.41)	269 (54.34)	120 (69.36)		
≤ 12 years	279 (41.89)	226 (45.66)	53 (30.64)		
Laboratory values (median) [IQR]					
GLDH 37 C (U/l)	3.3 [2.4]	3.4 [2.37]	2.9 [2.4]	0.363	567
ALT(GPT) 37 C (U/l)	23 [16.75]	23 [16]	25 [16]	0.233	378
AST(GOT) 37 C (U/l)	26 [11]	25.5 [11]	26 [11]	0.579	379
gamma-GT 37 C (U/l)	35.5 [52.5]	32 [37.75]	49 [83.75]	0.014*	462
alk.Phosphatase 37 C (U/l)	75 [47]	74 [46.5]	85.5 [46.25]	0.507	615
Hemoglobine (g/dl)	13.5 [2.3]	13.35 [2.2]	13.95 [2.5]	0.003*	2
Creatinine (mg/dl)	0.9 [0.34]	0.9 [0.35]	0.9 [0.32]	0.667	2
CRP (mg/l)	3.2 [7.77]	3.24 [8.3]	3.08 [6.74]	0.930	8
Total proteine (g/l)	69 [7.25]	69 [7.4]	68.8 [7.2]	0.893	17

N = number, SSQ = single sentence question, *=significant with p-value < 0.05, sd = standard deviation, ASA = American Society of Anesthesiologist Score, rCRI = revised cardiac risk index, NYHA = New York Heart Association Score, MoCA (Montreal Cognitive Assessment), IQR = interquartile range, GLDH = Glutamate dehydrogenase, ALT = Alanine aminotransferase, AST = Aspartate aminotransferase, GGT = gamma glutamyl transpeptidase, alk. Phosphatase = alkaline phosphatase, CRP = C-reactive protein

Of all 375 patients that answered the SSQ in the negative 56.05% scored ≥ 1 point on the AUDIT-C (Table 3). Most of them reached a maximum of 3 points on the AUDIT-C (74.87%, Table 3), but 94 (25.13%) scored more than 3 points on the AUDIT-C (Table 3). Of those patients 11.23% reported moderate alcohol intake (AUDIT-C = 4), and 13.90% scored 5 points or more, indicating frequent or high alcohol consumption (Table 3). In total, one quarter (25.13%) of the patients who denied daily alcohol consumption on the SSQ but scored positive on the AUDIT-C reached more than 3 points indicating moderate to high alcohol intake.

**Table 3:** Distribution of AUDIT-C results in patients that answered the SSQ in the negative

	All Patients (% of all patients with neg. SSQ and pos. AUDIT-C)	Women (% of women with neg. SSQ and pos. AUDIT-C)	Men (% of men with neg. SSQ and pos. AUDIT-C)	misssing
Negative SSQ and positive AUDIT-C (≥ 1)	375	115 (30.75)	259 (68.98)	1
Low or infrequent consumption (%)	281 (74.87)	101 (87.80)	178 (68.73)	2
AUDIT-C = 1 (%)	93 (24.87)	46 (40.00)	47 (18.15)	0
AUDIT-C = 2 (%)	93 (24.60)	31 (26.96)	61 (23.55)	1
AUDIT-C = 3 (%)	95 (25.40)	24 (20.87)	71 (27.41)	0
Significant consumption (%)	94 (25.13)	14 (12.18)	80 (30.89)	
Moderate alcohol consumption (%)				
AUDIT-C = 4 (%)	42 (11.23)	9 (2.41)	33 (12.74)	0
Frequent and/or high consumption (%)	52 (13.90)	5 (1.34)	47 (18.15)	
AUDIT-C = 5 (%)	26 (6.95)	5 (1.34)	21 (8.11)	0
AUDIT-C = 6 (%)	10 (2.67)	0	10 (3.86)	0
AUDIT-C = 7 (%)	11 (2.94)	0	11 (4.25)	0
AUDIT-C = 8 (%)	3 (0.80)	0	3 (1.16)	0
AUDIT-C = 9 (%)	2 (0.53)	0	2 (0.77)	0

AUDIT-C = Alcohol Use Disorder Identification Test- Consumption, SSQ = Single Sentence Question

Of 83 patients that answered the SSQ in the affirmative four scored 0 points on the AUDIT-C. The characteristics of those four patients are displayed in Table 4. We compared this small group of patients to the other patients that answered the SSQ in the positive, but the differences were not significant with the exception of the creatinine value that was higher in the four patients with contradicting answers (Table 4). It has to be mentioned that this small group had a lower mean MoCA test result than that of all other groups.

**Table 4:** Comparison of all patients that confirmed daily alcohol consumption in regards to their alcohol consumption according to the AUDIT-C

	All AUDIT-C patients	AUDIT-C 0–3 SSQ pos	AUDIT-C > 3 SSQ pos	missings
Number				
Age(mean ± sd)	71.24 (± 7.6)	68 (± 10.8)	71.41 (± 7.46)	0
Sex	83	4	79	0
Female, n (%)	16	2	14	
Male, n (%)	67	2	65	
ASA, n (%)				2
1	3	0	3	
2	27	1	26	
3	50	3	47	
4	1	0	1	
5	0	0	0	
Surgical department, n (%)				0
cardiac surgery	22	2	20	
Thoracic surgery- lung etc., esophagus	1	0	1	
Abdominal surgery	13	1	12	
Vascular surgery	3	0	3	
Orthopedic surgery	24	0	24	
others	20	1	19	
rCRI, n (%)				2
rCRI 1	35	2	33	
rCRI 2	23	0	23	
rCRI 3	18	1	17	
rCRI 4	5	1	4	
NYHA, n (%)				2
NYHA I	34	3	31	
NYHA II	32	0	32	
NYHA III	14	1	13	
NYHA IV	1	0	1	
MoCA Sum(mean ± sd)	23.95 (± 3.95)	22.5 (± 5.74)	24.03 (± 3.87)	0
Min - max values	14–30	15–27	14–30	
MoCA Education				
> 12 years	60	2	58	
≤ 12 years	23	2	21	
Laboratory values (median) [IQR]				
GLDH 37 C (U/l)	2.9 [2.93]		2.9 [2.93]	71
ALT(GPT) 37 C (U/l)	23.5 [16.75]	36.5 [18.5]	23.5 [16.25]	45
AST(GOT) 37 C (U/l)	24.5 (10.75]	49 [17]	23.5 [8.5]	47
gamma-GT 37 C (U/l)	59.5 [100.25]	91 [0]	57 [104.5]	55
alk.Phosphatase 37 C (U/l)	118.5 [74.5]	120 [0]	117 [93]	75
Hemoglobine (g/dl)	13.75 [2.68]	12.35 [0.7]	13.85 [2.65]	1
Creatinine (mg/dl)	0.87 [0.24]	1.18 [0.18]	0.86 [0.25]	1
CRP (mg/l)	3.03 [6.73]	4.09 [4.82]	3.02 [6.73]	2
Total proteine (g/l)	68.8 [6.6]	69.95 [2.72]	68.8 [7.6]	2

N = number, SSQ = single sentence question, *=significant with p-value < 0.05, sd = standard deviation, ASA = American Society of Anesthesiologist Score, rCRI = revised cardiac risk index, NYHA = New York Heart Association Score, MoCA (Montreal Cognitive Assessment), IQR = interquartile range, GLDH = Glutamate dehydrogenase, ALT = Alanine aminotransferase, AST = Aspartate aminotransferase, GGT = gamma glutamyl transpeptidase, alk. Phosphatase = alkaline phosphatase, CRP = C-reactive protein

According to the SSQ 127 patients (11.72%) drank alcohol daily. The percentage of patients consuming alcohol daily decreased with increasing age (Supplemental Material Table 1) and the percentage of daily drinkers was higher among men in all age groups (Supplemental Material Table 2).

Of all patients, that answered the AUDIT-C questionnaire, 32.04% scored 0 points indicating alcohol abstinence in the year before their elective surgery (Supplemental Material Table 2). Infrequent or low alcohol intake (AUDIT-C score of 1–3 points) was reported by 42.07% of the patients, while 25.90% reported a moderate to high alcohol consumption (4–12 points) (Supplemental Material Table 2).

## Discussion

This sub-analysis within the PROPDESC study aimed to evaluate the comparative reliability of two short methods to assess preoperative alcohol consumption among older surgical patients as an early, time-saving, and accurate detection of patients with AUD (Alcohol use disorder) is very important in a clinical setting. Important aspects for acceptance of a screening tool for alcohol consumption in the daily clinical setting beside its easy administration are difficulty, extensiveness, and suitability for self-completion. Assessment of a patient´s alcohol consumption level should be as early before surgery as possible, as this provides the opportunity to encourage preoperative alcohol reduction. Initiated early enough alcohol reduction could lead to a better postoperative outcome by improving several organic dysfunctions and in consequence reducing postoperative morbidity [7]. Discontinuation of alcohol consumption four to eight weeks prior to any surgical procedure could potentially decrease the incidence of postoperative complications [24].

In the immediate clinical setting the early and accurate detection of patients with alcohol drinking problems can help to avoid the occurrence of an AWS (alcohol withdrawal syndrome) [5].

When choosing a screening tool for alcohol consumption in the daily clinical routine, it is important to consider the time that both, physician and patient, are willing and able to provide for this procedure. Time-consuming assessments can strain both the respondent’s willingness and capacity to provide complete and accurate responses [25]. Surgeons indicated, that they preferred the use of a clinical assessment to a screening questionnaire, due to lack of time, busy schedules, and lengthy consent forms for surgery [26, 27]. Additionally, anesthetists tend to hand out the screening questionnaire selectively to intoxicated patients or known chronic high level drinking patients [26].

Additionally, detection of risky alcohol intake needs to be accurate taking into consideration that reliable testimony about alcohol consumption could depend on the setting in which the interview is posed. A person to person interview regarding a sensitive topic like alcohol consumption can lead to evasive answers. Kip et al. found that the prevalence rate of AUD determined by anesthesiologists was 6.9% compared to 18.1% if AUD was assessed using a computerized version of the AUDIT [28]. Inaccurate assessment of alcohol consumption may lead to the selective identification of individuals with severe alcohol dependency while overlooking patients who drink above recommended limits [26].

The results of our study show, that patient´s responses can differ considerably depending on how alcohol use is addressed during the routine pre-clinic visit prior to hospital admission. Of the patients that negated the SSQ about daily alcohol consumption and scored positive on the AUDIT-C questionnaire, 25.1% reported moderate (11.2%) to high (13.9%) alcohol consumption.

Significantly more men than women confirmed daily alcohol consumption (*p* < 0.001). This is in accordance with other surveys and studies about alcohol consumption [1, 21, 29–32]. The lower rate in our study sample could be influenced by the fact that it comprised of older patients.

Education level was significantly higher in patients that confirmed daily alcohol consumption (*p* = 0.0017) and scored moderate to high on the AUDIT-C (*p* = 0.003). A Danish study in middle-aged men and women found no significant differences of the alcohol consumption groups in age, but a significantly lower education in abstinent study participants [32]. The higher educational level of patients confirming daily alcohol intake (*p* < 0.001) and patients with an AUDIT-score > 3 (*p* = 0.003) could be an explanation for the fact that those patients also scored significantly higher in the MoCA test (*p* = 0.017 and *p* = 0.011, respectively).

The AUDIT-C has proven its sensitivity and specificity in various different settings and appears to be as good as if not better than the AUDIT [33] and national guidelines recommend the AUDIT questionnaire as a screening tool of at-risk alcohol consumption, harmful use or alcohol dependence and suggest the use of the AUDIT-C if the AUDIT is too complex or time is limited [34, 35].

An entity that has to be considered in the decision for a screening tool to accurately detect alcohol consumption in older patients is the possible presence of MCI (Mild Cognitive Impairment). According to Nasraddine et al. a result < 26 in the MoCA test could indicate a mild cognitive impairment [36]. The mean sum in the MoCA test was 23.00 in our study group with a mean age just above 72 years. This indicates that the presence of MCI has to be seriously considered in this patient group. Self-reports of older respondents (+ 70 years of age) with reduced working memory capacity are particularly affected by increased question difficulty [37]. Therefore, short questions and helpful explanations in a setting that provides enough time for the answers might help to evaluate information about alcohol consumption correctly. Paper-based or electronic versions of the AUDIT-C could be handed out to the patients before their face-to-face visit with the anesthesiologist in order to give the patient enough time to answer the questions.

The AUDIT-C could be completed by most patients without additional guidance, enables the treating physician to add important information to the patient’s pre-operative condition, and could be easily provided and completed paper-based or electronically. The use of electronic devices such as a computer or tablet has many advantages. It is cost-effective as it saves valuable face-to-face time with the anesthesiologist for personal questions regarding the upcoming surgery. Questionnaires could be programmed to allow only valid and consistent responses [25]. If necessary pictures of beverage containers or explanations could be included that facilitate the understanding of standard drink sizes further [38]. Patients that are unable or unwilling to use an electronic device can fill in a paper-based version of the AUDIT-C during their pre-clinic assessment visit, maybe assisted by an interviewer that can provide motivation and clarification, but their presence may negatively affect the respondent´s willingness to answer sensitive questions [25].

To support the results of alcohol consumption questionnaires further blood biomarkers could be taken into consideration. Assessment of current levels of intoxication might include the direct biomarkers Blood Alcohol concentration (BAC), phosphatidyl ethanol (Peth), and fatty acid ethyl ester (FAEE) [39]. The indirect alcohol biomarkers, such as MCV (mean corpuscular volume), AST (aspartate aminotransferase), ALT (alanine aminotransferase), GGT (gamma glutamyl transpeptidase), and CETP (cholesteryl ester transfer protein), could indicate heavy alcohol use indirectly, as they are mainly correlated to the impact of chronic alcohol use on the liver and red blood cells [39]. One has to bear in mind that they are greatly influenced by factors, such as age, sex, and/or organ damage [40]. As the laboratory values of the PROPDESC study were collected to screen for possible POD-predicting indicators the availability of the indirect alcohol biomarkers for additional evaluation were very limited. Still we found a difference in GGT value in the SSQ sample as well as the AUDIT-C sample with patients reporting higher amounts of alcohol consumption having significantly higher values of GGT (*p* < 0.001). Even though GGT is widely used as a biomarker for sustained excessive alcohol intake [39], its specifity is reduced with comorbid medical conditions not related to alcohol (e.g. nonalcoholic liver diseases, nephrotic syndrome, and pancreatitis [41]). Even though high MCV may indicate excessive drinking, we did not include it in our analysis as it is neither sensitive nor specific for alcohol use and factors such as age, sex, and pre-existing conditions can influence the results [39]. Furthermore, the following limitations associated with the use of blood biomarkers for alcohol use have to be considered: firstly, currently used biomarkers could differentiate between excessive alcohol use and abstinence, but lack precise quantitative information about the amount of consumption [39]. Blood biomarkers could not detect detailed drinking patterns and their sensitivity and specificity is influenced by comorbid health problems [39].

Limitations: The study excluded younger patients and patients with difficulties in the German language or those with pre-existing cognitive impairments like dementia. While this is understandable for the accuracy of the study, it limits the inclusivity of the sample and may not fully reflect the alcohol consumption patterns in these excluded populations. The PROPDESC study was conducted in a mono-centric setting. This limits the generalizability of the results to other populations or regions, as different hospitals may have different patient demographics, healthcare practices, and cultural contexts regarding alcohol use.

Both assessments were done by study personnel composed of Study Nurses and medicinal students in the context of the PROPDESC study. In such a scenario, there could be a response bias since the desire to portray a favorable image significantly influences self-reported alcohol consumption [42]. Furthermore, the study assessment was conducted apart from the anesthesiological evaluation. Patients might be more honest about their drinking habits when asked by their surgeon or anesthesiologist, because they might be more aware of the relevance of a correct answer.

## Conclusions

The method of questioning influences the accuracy of the information older patients provide about their alcohol consumption during pre-clinic visits. A single sentence question about daily alcohol consumption could fail to identify patients with possibly health- and surgery-relevant alcohol consumption levels and anesthesiologist might miss the opportunity to implement necessary prevention measures to avoid AWS in patients with severe AUD. The AUDIT-C could evaluate the patient´s alcohol intake more precisely than a single sentence question without the need of considerably more time as it could be easily self-administered. It should therefore be added to the routine assessment at the pre-hospital visit of the patient. The AUDIT-C is a suitable tool to assess a patient´s alcohol consumption level. Additionally, answering a questionnaire provides initiative to the patient and a more objective assessment to the physician. Furthermore, it could relieve both from sensing a moral evaluation of the patient´s alcohol consumption level.

## Electronic supplementary material

Below is the link to the electronic supplementary material.

Supplementary Material 1

## Data Availability

No datasets were generated or analysed during the current study.

## Abbreviations

WHO: World Health Organisation
PROPDESC-study: Pre-Operative Prediction of postoperative delirium by appropriate Screening-study
APC: Per capita alcohol consumption
EU: European Union
AUDIT-C: Alkohol Use Disorder Identification Test-Consumption
AUDIT: Alkohol Use Disorder Identification Test
AWS: Alcohol Withdrawal Syndrome
ASPSTF: The U.S. Preventive Service Task Force
NIAAA: National Institute on Alcohol Abuse and Alcoholism
SASQ: Single Alcohol Screening Question
SSQ: Single sentence question
POD: Postoperative delirium
ASA: American Society of Anesthesiologists class
NYHA: New York Heart Association
rCRI: Revised cardiac risk index
MoCA: Montreal Cognitive assessment
AUD: Alcohol Use disorder
GGT: Gamma Glutamyl Transpeptidase
MCI: Mild Cognitive Impairment
