# Supplementary material for: Preoperative evaluation of alcohol consumption in older patients
Source: Addict Sci Clin Pract. 2025 May 21;20:44. doi: 10.1186/s13722-025-00569-8 (PMC12093839; doi:10.1186/s13722-025-00569-8)
Supplement: Supplementary file 1 — Supplementary Material 1 [file 13722_2025_569_MOESM1_ESM.docx]

Supplemental Material:

Preoperative evaluation of alcohol consumption in older patients

Vera Guttenthaler^1*^, Maria Wittmann^1^, Jan Menzenbach^1^

1 University Hospital Bonn, Venusberg-Campus 1, 53127 Bonn, Germany

Table 1: Alcohol consumption in age and gender groups according to SSQ

|  | All patients n, (%) | Women 60-69 years  (n, %) | Women 70-79 years  (n, %) | Women ≥ 80 years  (n, %) | Men 60-69 years  (n, %) | Men 70-79 years  (n, %) | Men ≥ 80 years  (n, %) | missing |
| --- | --- | --- | --- | --- | --- | --- | --- | --- |
| No  daily alcohol consumption | 957 (88.28) | 133 (13.90) | 190 (19.85) | 81 (8.46) | 229 (23.93) | 226 (23.62) | 95 (9.93) | 3 |
| Daily alcohol consumption | 127 (11.72) | 9 (7.09) | 7 (5.51) | 6 (4.72) | 49 (38.58) | 41 (32.28) | 15 (11.81) | 0 |

n = number of patients

Table 2: AUDIT-C results in relation to age and sex

|  | All patients (%) | Women 60-69 years | Women 70-79 years | Women  ≥ 80 years | Men  60-69 years | Men  70-79 years | Men  ≥ 80 years | missings |
| --- | --- | --- | --- | --- | --- | --- | --- | --- |
| Total (n, %) | 668 (100.00) | 89 (13.32) | 110 (16.47) | 53 (7.93) | 185(27.69) | 160 (23.95) | 69 (10.33) | 2 |
| AUDIT-C  0 points (n, %) | 214 (32.04) | 43 (6.44) | 51 (7.49) | 28 (4.19) | 36 (5.39) | 36 (5.39) | 19 (2.84) | 1 |
| AUDIT-C  1-3 points (n, %) | 281 (42.07) | 37 (5.54) | 47 (7.04) | 18 (2.69) | 71 (10.63) | 78 (11.68) | 29 (4.34) | 1 |
| AUDIT-C  ≥ 4 points (n, %) | 173 (25.90) | 9 (1.35) | 12 (1.80) | 7 (1.05) | 78 (11.68) | 46 (6.89) | 21 (3.14) | 0 |
| highest AUDIC-C score | 12 | 10 | 6 | 5 | 12 | 10 | 10 |  |

AUDIT-C = Alcohol use disorder identification test on consumption, n = number of patients
